# Driving Consumer Value Co-creation and Purchase Intention by Social Media Advertising Value

**DOI:** 10.3389/fpsyg.2022.800206

**Published:** 2022-02-24

**Authors:** Ali Hussain, Ding Hooi Ting, Muhammad Mazhar

**Affiliations:** Department of Management and Humanities, Universiti Teknologi PETRONAS, Seri Iskandar, Malaysia

**Keywords:** social media advertising, value co-creation, purchase intention, service-dominant logic (S-D logic), e-WOM (electronic word-of-mouth)

## Abstract

Social media advertisement (ad) is a growing phenomenon designed to reach and engage customers. However, despite their continued adoption, less remains known regarding the effectiveness of social media ads to co-create brand value. In response to this gap, this study aims to deepen the theoretical understanding of consumer value co-creation through social media advertising value. The data were collected using purposive sampling from 286 experienced social-media users, and the model was tested using partial least square (PLS)-based structural equation modeling. The results indicate that entertainment, aesthetic appeal, interactivity, and trendiness significantly affect the adverting value of social media ads. In turn, ad value affects consumers’ intention for value co-creation. Consequently, our findings suggest the importance of social media advertising value where marketers may enhance consumer-brand engagement (CBE) by incorporating interesting content, which may encourage the customer’s interaction with the social media ads and strengthen value co-creation behavior. The results further contribute to nascent marketing literature by demonstrating that value co-creation acts as an antecedent to generating positive electronic word-of-mouth (e-WOM) on social media platforms to drive consumers’ online brand purchase intention.

## Introduction

Digital marketing has transformed consumer behavior rapidly. It has been argued that customers are co-creating the brand value by coming up as active participants and collaborators that generate new ideas rather than just passive observers (Barile et al., 2021). Brands are using social media tools to create opportunities for consumers to engage actively (e.g., live sessions and feedback polls) and create value for both parties (Cheung et al., 2021). Out of these frequently used online customer engagement techniques, social media advertising is getting significant attention as it involves interactive marketing that facilitates consumer–brand interactions, driving value co-creation (Lin et al., 2018). Traditional advertising was considered only as a one-way medium of communication to bring awareness about newly launched features of products and services. However, with rapid web 4.0 technological advancements and interactive social media features, social media advertising becomes a means to exchange valuable information, medium of interaction, and allows customized customer-generated content through online customer feedback (Schivinski and Dabrowski, 2016; Saura et al., 2021).

Notwithstanding, the fact that rapid technological advancement enables firms to develop interactive “engagement ads” where consumers are willing to collaborate and exchange resources, leading to value co-creation and CBE opportunities (Cheung et al., 2021; Saura et al., 2021). To date, most prior studies mainly focused on the positive effect of social media advertising value on consumers’ attitude (Hamouda, 2018), brand attitude (Langaro et al., 2018), flow experience (Cuevas et al., 2021), and purchase intention (Lee and Hong, 2016). Thus, it is crucial for the brands to not only focus on how advertising works but also how consumers work with advertising (Nyström and Mickelsson, 2019). Additionally, since the brand value is jointly created by both marketers and consumers, the consumer-advertising relationship deserves more attention and should be investigated from a co-creation standpoint.

In response to this gap, this study aims to explore the underlying factors, specifically entertainment, aesthetic appeal, interactivity, and trendiness, as an indicator of the social media advertising value to promote value co-creation and consumers’ positive word-of-mouth (WOM). Through the lens of service-dominant logic (S-D logic), the finding by Vargo et al. (2008) proposed that consumers are important actors in co-creating the value, and based on this, determine how they experience the service. Given the highly interactive nature of social media advertising, S-D logic may be a better fit for evaluating the customer experience with social media ads than traditional approaches (Lin et al., 2018). For example, Rathore et al. (2016) have emphasized that social media activities are a new medium of communication to create/co-create value among customers. Similarly, Abeza et al. (2020) have explored the theoretical importance of social media advertisement (ad) value where customers see the ad as a source of entertainment and information-seeking platform. Considering these developments, this study contributes to the nascent marketing and advertising literature by investigating the role of effective social media advertising from a value co-creation perspective. More specifically, to fill this gap in the literature, this study proposes a parsimonious model that illuminates the antecedents and consequences of value co-creation with social media in ad-to-consumer (A2C) perspective based on the S-D logic. In this endeavor, the research aims to address the following research objectives:

1. Examine the impact of social media advertising value on consumers’ value co-creation behavior.
2. Explore the behavioral consequences of value co-creation in the A2C context.

The significance of the study is twofold. First, this research will conceptualize the “social media advertisement value and co-creation framework” for the brands to enhance customers’ brand interaction through a collaboratively value-created communication in the A2C context. Second, this research also responds to a recent call for research on interactive social media advertising strategy. For brand managers and advertisers, our findings have more relevant guidelines and implications by understanding how the consumer perception of social media brand advertising could help to learn more about how ads create value for customers. Additionally, this might also help brands to rethink and redesign their social media advertising activities to co-create value with the customers for a competitive mark in the online branding market.

## Literature Review

### Service-Dominant Logic and Value Co-creation

The concept of dominant logic in the service marketing literature is evident when Vargo and Lusch (2004) advocated the concept of goods-dominant logic. The studies reflect the products and commodities as the medium of exchange and value creation among the customers (Vargo and Lusch, 2008; Grönroos, 2011). The revolutionary studies in the marketing literature have focused on the exchange of goods and commodities (Shamim et al., 2016; Grönroos, 2017). However, there is increasing emphasis in the service marketing literature on the new dominant logic paradigm where customers are the active co-creators of the value (Svensson and Grönroos, 2008; Stampacchia et al., 2020). The phenomena give rise to a S-D logic paradigm where Vargo and Lusch (2004) have conceptualized the understanding of new reflection products and commodities as only a medium of exchange and value through the co-creation perspectives. Customers are the primary focus of attention, and they are treated as the main actor who is involved in the value co-creation perspective (Vargo and Lusch, 2016; Shamim et al., 2017). S-D logic further advocates that value co-creation only originates if the customers are taken as coproducers of the value (Sarmah et al., 2018). To unravel the rapidly changing world view of marketing and value co-creation among customers, this study has conceptualized the social media customers’ value co-creation framework through the lens of the S-D logic paradigm. The conceptualization aims to examine the role of social media advertising to engage the customers toward value co-creation (Sweeney et al., 2018). The rapidly changing consumer behavior advocates the need to study the advertising value-driven factors to enhance customers’ value co-creation which significantly affects the electronic word-of-mouth (e-WOM) and uplifts the customers’ purchase intentions toward the specific service and brand.

## Conceptual Framework and Research Hypotheses

### Entertainment

Advertisement-related entertainment is defined as the capability of the ad to fulfill the viewers’ desire for aesthetic enjoyment, escapism, diversion, or emotional release (Martí Parreño et al., 2013; Martins et al., 2019). Prior studies suggested that a more entertaining and pleasurable ad can grab the viewers’ attention, and these features can be used to enhance the involvement of customers and make them more acquainted with the advertised product or service (Haghirian and Madlberger, 2005). Additionally, research on social media advertising aligns with this prediction that more pleasurable, enjoyable, and humorous elements of the ad are positively related to perceived ad value (Ducoffe, 1995; Abbasi et al., 2021). Thus, we proposed the following hypothesis:

**H1:** The perceived entertainment of social media ads is positively associated with perceived ad value.

### Aesthetic Appeal

Advertisement-related aesthetic refers to ad impressive aspects, including colors, themes, sound, or music, which are closely related to the user’s ad experience (e.g., by maintaining user interest in human-computer interactions) (Bhandari et al., 2019). For example, Tuch et al. (2009) illustrated that visually appealing stimuli motivate users to cognitively clarify and comprehend the visual object. Extant literature suggests that users respond toward the products based on their aesthetic attributes and combinations (e.g., dynamic themes used, luminance, patterns, and shapes) (Moshagen and Thielsch, 2010; Bhandari et al., 2019). In the context of advertising, Abbasi et al. (2020) and Kusumasondjaja and Tjiptono (2019) showed that aesthetic appeal enhances customers’ perception of ad quality and favorably influences their behavioral intention. Visual appeal affects users’ opinions and preferences toward a wide range of objects, such as web pages, ads, and physical products (Lavie and Tractinsky, 2004). Likewise, aesthetics in social media ads contributes to enhanced viewers’ attention and results in a greater inclination to interact with ads and thus enhance ad-related value. Based on this rationale, we deduced the following hypothesis:

**H2:** The perceived aesthetic appeal of social media ads is positively associated with perceived ad value.

### Interactivity

Brand-consumer communication is altering the interactive aspects of social media advertising, and consumers are playing a more influential role in the ad experience. Interactivity is defined as a two-way conversation or dialogue between the user and brand through the online channel that augments the feeling of immediacy and closeness (Hidayanti et al., 2018). Similarly, in the advertising context, interactivity aims to give end users the ability to communicate successfully as senders or receivers with brands through real-time to access or deliver information on-demand (Sreejesh et al., 2020). Bozkurt et al. (2020) found that marketers can get different opinions and perspectives through social media advertising and have a place to talk and trade ideas with the end users. Therefore, it is argued that customers’ demands and requirements and their thoughts and suggestions on the product and brand may be obtained in real-time by using social media ads as an interactive communication channel between the brands and consumers. Thus, we purposed the following hypothesis:

**H3:** The perceived interactivity of social media ads is positively associated with perceived ad value.

### Trendiness

Trendiness represents the extent to which consumers perceive that information disseminated about the brand through social media ads are the latest and up-to-date (Ramadan et al., 2018; Cheung et al., 2021), including new features, trends, hot themes, or compatibility concerning the brand. For example, Cheung et al. (2020a) demonstrated that users are immersed in the brand itself and brand-related trendy informational ad content (e.g., current hot topics) that piques their interest. Brands often update their ads on the different social media platforms to stay current, sharing the latest news about the company, such as product innovations and new products (Dessart et al., 2015; Ramadan et al., 2018). Similarly, compared with traditional ads, consumers increasingly rely on online social media platforms to obtain more useful and up-to-date brand-related information. We proposed the following hypothesis:

**H4:** The perceived trendiness of social media ads is positively associated with perceived ad value.

### Advertisement Value

H1–H4 explore the effect of specific social media ads on overall users’ perceived ad value. To obtain a comprehensive overview of the social media ads and value co-creation, we then assessed the effect of perceived brand social media ad value on users’ co-creation experience. Ducoffe (1996) illustrated ad-related value as the subjective advertising evaluation of how worthy or valuable an ad is perceived to be. With the rapid proliferation of social-media communication, effective social media ads provide possibilities for companies to interact and communicate with customers, therefore improving consumers’ capacity to engage with businesses in a value co-creation process that is conducive to mutually enhanced perceived value (Godey et al., 2016; Singaraju et al., 2016). Brands are investing a growing sum in social media brand communities to better interact with their customers and uncover and support co-created innovation prospects (Rashid et al., 2019). Therefore, we expected those social media ads that offer higher user-perceived value are more likely to strengthen the interaction between consumers and brands, thus motivating consumers’ resource integration into co-creating brand meaning and value. We hypothesized the following:

**H5:** The perceived social media ad value has a positive effect on value co-creation.

### Value Co-creation, Electronic Word-of-Mouth, and Purchase Intention

After the 2000s, co-creation has emerged as a dominant logic paradigm where firms and the customers are the active coproducers of the value (Neuhofer and Buhalis, 2017; Shamim et al., 2021). It has been observed that those firms actively initiating the engagement platforms for the customers to exchange the service ideas end up with a positive brand image (Ibrahim et al., 2017). The online image of the products/services relies on many factors, where one of the essential factors is e-WOM (Serra-Cantallops et al., 2018; Meilhan and Economics, 2019). The online context of shopping is quite different from the physical context where customers rely on prior users’ feedback of similar products/services (Tong, 2010; Pizzi et al., 2019). Thus, value co-creation is an important mechanism to engage the potential customers prior to using the services (Kunja and Acharyulu, 2018; Graessley et al., 2019). The engagement strategies are competitive as they bring the customized demands of the customers (knowledge) to the online firms. The prior knowledge about customers’ demands enables the firm to produce a similar offering that will create a positive word of mouth and enhance the chances of purchasing similar products/services from the online brand through ad (Shin et al., 2014; Drugău-Constantin, 2019). We posited the following hypotheses:

**H6:** Customers’ value co-creation with the social media advertising value significantly impacts customers’ e-WOM.

**H7:** Customers’ value co-creation with the social media advertising value significantly impacts customers’ purchase intention.

### Electronic Word-of-Mouth and Purchase Intention

With the rapid proliferation of the Internet and the ubiquitous social media sites, the tendency to gain popularity among users has become more certain (Balaji and Roy, 2017). Contrary to traditional WOM, e-WOM communication offers an opportunity for users to get newfangled and real-life information from previously inaccessible sources (e.g., review sites, blogs, and social media sites) (Fan and Miao, 2012). Bastos and Moore (2021) illustrated that over four billion Internet users are currently exposed to hundreds of millions of reviews, comments, and tweets *via* blogs, review sites, and social networking platforms. By using social media platforms, the consumer can readily share and collect information related to products and services in a timely and cost-effective manner (Tien et al., 2019; Sun et al., 2021). According to a survey, 61% of consumers examine e-WOM before purchasing any product, and 80% of consumers are only willing to purchase online after reading customer opinions and recommendations (Yusuf and Busalim, 2018). Moreover, the impact of e-WOM is significant when it comes to the customer’s purchase intention because customers are more concerned about the online feedback of the other customers who have already used similar products. We proposed the following hypothesis:

**H8:** Customers’ e-WOM significantly impacts customers’ purchase intention.

## Methodology

### Measurement

This research measures eight variables comprising entertainment, aesthetic appeal, interactivity, trendiness, ad value, value co-creation, e-WOM, and purchase intention. Each of the constructs included in our model was adapted from existing multi-item measurement scales, with slight modifications to reflect our research context of social media ads. For example, three items of ad-related entertainment adapted from Cheung et al. (2020b). For aesthetic appeal, we gauged a three-item scale developed by Balaji and Roy (2017). To measure ad interactivity, we used a four-item scale developed by Nasir et al. (2021). To gauge trendiness, we deployed a two-item measure developed by Algharabat (2017). We modified a three-item scale from Martins et al. (2019) to gauge ad value. The five items of value co-creation were adapted from Cheung et al. (2021). The two items of e-WOM were measured and adapted from Cheung et al. (2021). Finally, a three-item scale of purchase intention was adapted from Cheung et al. (2020b). The questionnaire concluded with relevant demographic questions (e.g., gender, age, profession, and education) to facilitate the understanding of the sample characteristics. Items were measured using five-point Likert-type scales, ranging from 1 (“strongly disagree”) to 5 (“strongly agree”).

### Data Collection

An online survey generated *via* Google form was used to assess our proposed research model. Study participants aged 15 years and above were recruited from the Malaysian population. We used the G*power analysis to compute our sample size, which is widely recommended for structural equation modeling (SEM) (Hair et al., 2019). The poll link was shared on several social media channels to collect data. Respondents were encouraged to complete the online survey and share the information with others in their network. This process was repeated until we had attained the desired number of responses needed for analysis (Smit et al., 2020). Participants must be at least 15 years old and have an active social media account to be considered for this study. As a result, if a participant indicated that they were under the age of 15 and did not use social media, the survey was stopped with a thank you note on the screening question. After eliminating incomplete surveys, we retained 286 usable questionnaires for further analysis. Using the input parameters (*f*^2^ = 0.15, α = 0.05, power = 0.95, and predictors = 4) in G*power analysis (Faul et al., 2007), the minimum required sample size was 129, thus indicating the adequacy of our attained sample. An overview of the respondents’ demographic profiles is shown in Table 1. To test the hypotheses, we deployed partial least square (PLS)-based structural equation modeling (PLS-SEM) (Richter et al., 2020), which was implemented using SmartPLS 3.2.8 software. We then presented our findings pertaining to indicator reliability, convergent validity, and discriminant validity of the measurement model, followed by an assessment of the structural model.

**TABLE 1:** Respondent profile (*n* = 286).

Measure	Item	*N*	Percentage (%)
Gender			
	Female	164	57.3
	Male	122	42.6
**Age**			
	15–22	61	21.3
	23–30	98	34.2
	31–38	55	19.2
	39–46	47	16.4
	47 and above	25	8.74
**Education**			
	School	39	13.6
	Diploma	63	22.0
	Undergraduate	98	34.3
	Masters	63	22.0
	Ph.D.	23	8.04
**Profession**			
	Employed full time	69	24.1
	Employed part-time	56	19.5
	Unemployed	44	15.3
	Student	117	40.9
**Ethnicity**			
	Malay	98	41.5
	Chinese	71	25.2
	Indian	52	21.4
	Other	65	11.8
**Frequently used SNS**			
	Facebook	86	30.0
	Instagram	72	25.2
	YouTube	69	24.1
	Snapchat	38	13.3
	Others	21	7.3
**Frequency of viewing social media ads**			
	1–5 ads per day	64	22.3
	More than 5 ads per day	84	29.4
	1 ad in 2–3 days	51	17.8
	1 ad in 4–5 days	47	16.4
	1 ad in a week	40	13.9

## Data Analysis and Results

### Measurement Model Results

First, the measurement model was assessed to analyze the convergent validity by examining outer loadings of associated items for each construct, composite reliability (CR), and average variance extracted (AVE) (Hair et al., 2017). According to Henseler et al. (2016), the outer loading of associated items of each construct should be greater than the recommended value of 0.7. All the items have loading greater than the threshold value of 0.7 (Table 2). The second criterion to confirm the convergent validity is that the value of Cronbach’s alpha (CA) and CR also exceeded the recommended value of 0.7 (Henseler et al., 2009). Table 2 demonstrates that all the constructs have CA and CR values greater than 0.7. AVE criteria are also used to assess convergent validity, and the value of the AVE must be at least 0.5 which consider adequate to explain more than half of the variance of its indicators (Hair et al., 2013). As seen in Table 2, all the latent constructs have an AVE value of more than the recommended level of 0.5; therefore, all three conditions for convergent validity are fulfilled, so convergent validity is not an issue in this study.

**TABLE 2:** Measurement model assessment.

Construct	Items	Outer loading	Composite reliability	Cronbach’s alpha	AVE
Entertainment	ENT1	0.849	0.881	0.798	0.712
	ENT2	0.820			
	ENT3	0.862			
Aesthetic appeal	AP1	0.841	0.881	0.797	0.711
	AP2	0.845			
	AP3	0.843			
Interactivity	INT1	0.798	0.912	0.871	0.721
	INT2	0.859			
	INT3	0.842			
	INT4	0.894			
Trendiness	TRD1	0.895	0.899	0.775	0.816
	TRD2	0.912			
SM advertising value	AV1	0.840	0.896	0.827	0.743
	AV2	0.845			
	AV3	0.899			
Value co-creation	VC1	0.776	0.888	0.842	0.613
	VC2	0.773			
	VC3	0.796			
	VC4	0.795			
	VC5	0.902			
e-WOM	e-WOM1	0.914	0.897	0.770	0.812
	e-WOM2	0.889			
Purchase intention	PI1	0.874	0.920	0.878	0.793
	PI2	0.883			
	PI3	0.913			

Furthermore, the discriminant validity of the constructs was assessed by the heterotrait-monotrait (HTMT) ratio of correlation (Hair et al., 2020). HTML (with a cutoff ratio of 0.85) is considered to be a more emerging and conservative approach to examine the discriminate validity (Henseler et al., 2015). Criteria of discriminant validity were also achieved as all the values were below the threshold level of 0.85 (Table 3).

**TABLE 3:** Discriminant validity analysis.

	ADV	AP	ENT	e-WOM	INT	PI	TRD	VC
Advertising value								
Aesthetic appeal	0.486							
Entertainment	0.444	0.6						
e-WOM	0.467	0.377	0.351					
Interactivity	0.505	0.442	0.376	0.418				
Purchase intention	0.483	0.402	0.438	0.355	0.413			
Trendiness	0.435	0.444	0.382	0.401	0.481	0.39		
Value co-creation	0.317	0.467	0.522	0.353	0.488	0.664	0.574	

### Structural Model Results

**FIGURE 1:**
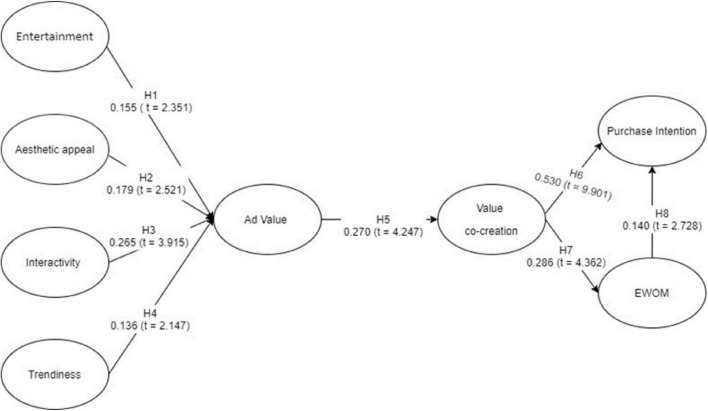
Partial least square (PLS)-based structural equation modeling (PLS-SEM) model specification for measurement model assessment.

After satisfactory results of the measurement model, the next step is to test the structural model. Bootstrapping of 5,000 resamples was used in this study to examine statistical significance of path coefficients, effect size, and *T*-value (Rezaei et al., 2018). The results show that all the hypotheses, i.e., H1–H8, are supported by the data (refer to Table 4 and Figure 1). The hypothesis of entertainment (path = 0.155; *p* < 0.05 and *T* = 2.351), aesthetic appeal (path = 0.179; *p* < 0.05 and *T* = 2.521), interactivity (path = 0.265; *p* < 0.05 and *T* = 3.915), and trendiness (path = 0.136; *p* < 0.05 and *T* = 2.147) significantly enhance the perceived value of social media ads, whereas, perceived social media ad has strong and significant effect on value co-creation (path = 0.270; *p* < 0.05 and *T* = 4.247). However, value co-creation has a significant positive effect on purchase intention (path = 0.530; *p* < 0.05 and *T* = 9.901) and e-WOM (path = 0.286; *p* < 0.05 and *T* = 4.362). Finally, e-WOM significantly impacts consumer purchase intention (path = 0.140; *p* < 0.05 and *T* = 2.728).

**TABLE 4:** Structural model assessment.

Hypothesis	Path	Path coefficient	SE	*f* ^2^	*T*-Value	*p*-Value	Results
H1	ENT → ADV	0.155	0.066	0.025	2.351	0.019	Supported
H2	AP → ADV	0.179	0.071	0.031	2.521	0.012	Supported
H3	INT → ADV	0.265	0.068	0.077	3.915	0.000	Supported
H4	TRD → ADV	0.136	0.063	0.021	2.147	0.032	Supported
H5	ADV → VC	0.270	0.064	0.079	4.247	0.000	Supported
H6	VC → PI	0.530	0.054	0.392	9.901	0.000	Supported
H7	VC → e-WOM	0.286	0.066	0.089	4.362	0.000	Supported
H8	e-WOM → PI	0.140	0.051	0.028	2.728	0.006	Supported

## Discussion and Implications

Given the limited attention afforded to consumers’ social media-related behaviors in building a customer–brand relationship and value co-creation, especially with respect to social media advertising, this study examines the role of several ad-value-related drivers in driving consumers’ value co-creative behavior and purchase intention. The advertising context is changing from traditional means of only communicating the information toward engaging customers for the valuable outcome (Wu and Li, 2018; Fraccastoro et al., 2021). The customers are demanding something more than a traditional ad that engages their attention, gauges their emotional captivity, and involves them in the information about their brands of interest on social-media ads (Willemsen et al., 2018). The role of customers has changed from passive recipients of the value to active co-creators of the value (Siddique et al., 2021; Smith et al., 2021). Brands can only succeed by providing a platform where customers can share their opinions and feedback about brand offerings and encourage users to create, share, and like similar interactive brand content with other users (Chwialkowska, 2019). This can be accomplished by taking digital advertising as a service perspective, service as logic, or a perspective on value creation, rather than a type of offering.

This study contributes to the current literature by presenting a comprehensive framework that illustrates how social media advertising antecedents drive value co-creation behavior which further leads to enhanced e-WOM and purchase intention. Overall, our results validate the finding of the previous studies, indicating that the four dimensions of entertainment, aesthetic appeal, interactivity, and trendiness, of social media advertising, reflect a holistic experience within social media ads (Ducoffe, 1996; Tran et al., 2020; Hussain et al., 2021). As expected, social media advertising value dimensions significantly enhance consumers’ intention to engage with social media advertising and enhance consumers’ involvement in the co-creation process. For instance, interactivity has the strongest impact on enhancing social media advertising value. People increasingly use social media platforms due to their highly interactive nature that allows consumers to socialize and network better (Tan et al., 2019). Arguably, the interactive nature of advertising, such as surveys, quizzes, and games, that encourages consumers to engage with ads tends to generate favorable consumer responses (Harmeling et al., 2017). Then, the significant effect of aesthetic appeal on perceived advertising value is remarkably meaningful as findings from prior studies suggested that aesthetic elements play a vital role in affecting consumers’ preferences and behavior toward a wide range of things such as web pages, advertising, physical products, and packages (Jiang et al., 2016; Balaji and Roy, 2017). This means that aesthetic appealing aspects of the advertising such as intricate textures, color variations, and dissimilar shapes or sizes enhance customers’ ability to engage with the ad and therefore provoke higher perceived advertising value (Hussain et al., 2021). Similarly, our results also showed that ad-related entertainment exerts a significant, positive effect on users’ perceived advertising value of social media ads (Martins et al., 2019). That is, most people use different social media platforms to satisfy their need for escapism, aesthetic enjoyment, and pleasure (Mahmoud, 2015; Kim, 2020). Therefore, consumers pay more attention to the ads that fulfill their entertainment-seeking motives and thus enhance the perceived value of the ad. In line with the findings from the prior studies, a significant positive result was found between the trendiness and perceived advertising value (Algharabat, 2017; Firat, 2019). This finding underscores the significance of disseminating the latest and trendiest information related to the brands, for example, product benefits, compatibility features, and safety guidelines that help the consumer to understand the product and how to maximize their use (Van-Tien Dao et al., 2014; Maseeh et al., 2021).

Furthermore, in line with the finding of prior sties, our results demonstrate that effective social media advertising serves as a crucial venue for consumer-brand social interchange, strengthening consumers’ desire to engage in the ongoing value co-creation process (Spurgeon, 2015; Orazi et al., 2016). In other words, by using effective social media advertising brands can involve consumers in co-create brand value, by offering solutions for brands to solve identified problems, provide recommendations to improve the quality of existing products, or become involved in the brands’ new product development process (Nysveen and Pedersen, 2014; Cheung et al., 2020b).

Finally, this study has highlighted the importance of e-WOM where customers are most likely to provide feedback against specific ads they are receiving online (Belanche et al., 2020). The customers’ feedback is of significant importance as most customers rely on online feedback before going for the actual purchase. Therefore, brands need to engage their customers positively through social media advertising which subsequently generates positive WOM (Loureiro and Sarmento, 2019). This positive WOM shall increase the customer’s intentions to purchase the services communicated through social media ads.

### Theoretical Implications

This study makes several theoretical contributions. First, this study departs from the prior studies on the co-creation process through social media advertising, which is mainly conceptual (de Oliveira and Cortimiglia, 2017) and qualitative (Aaltonen, 2010) in nature. Regardless of the increasing importance of co-creation of value, empirical inquiry into the A2C perspective is still nebulous. Based on this, this research addresses this gap in the nascent co-creation and advertising literature by empirically examining advertising as an element in the service process. As Vargo et al. (2017, p. 49) illustrated, the definition of service as “resources applied for the benefit,” perceived valuable social media advertising can thus be viewed as a consumer’s initial or early brand-related value-creating vehicle, rendering these social media advertising an integral part of consumers’ advertising-as-a-service experience.

Second, this study develops and validates a compressive research model of customer value co-creation with social media advertising. To the best of our knowledge, very limited prior studies apply the S-D logic to understand the customer’s value co-creation with social media advertising (Hidayanti et al., 2018; Zadeh et al., 2019). Therefore, we extended and validated empirically a sparse but compressive model that demonstrates how different social media advertising value-related elements, entertainment, aesthetic appeal, interactivity, and trendiness influence consumers’ value co-creation behavior which ultimately driving e-WOM and repurchase intention (Choi et al., 2016; Ribeiro-Navarrete et al., 2021). Consequently, this research provides a novel viewpoint that to keep the attention of consumers, advertising must provide the consumers with something they desire while also driving the consumers’ value-creation activities in some way.

### Managerial Implications

From a managerial perspective, this study offers several practical implications for marketing managers and advertisers to use social media advertising as a viable source of customer-brand interaction to strengthen consumers’ value co-creation intention. Rather than using social media advertising as just another way to reach customers, see it as a valuable instrument that encourages consumers to be active participants in the value co-creation process and ultimately encourages them to spread e-WOM voluntarily.

We identified that a significant impact of ad interactivity on overall user-perceived ad value illustrates that consumers value social media ads, which encourage them to share their ideas and feedback in the overall brand value co-creation process. We thus recommended the designers of social media advertisers to develop such interactive ads that initiate competitions to entice consumers to leave their suggestions and ideas for improvements to existing items or new product innovations to compete in exchange for rewards that may result in improved customer-brand interaction. It is also recommended that marketers seek to enhance the social media ad-related value by an appropriate combination of aesthetic appeal (e.g., colors, music, and themes). Aesthetics are not used as means of pleasure and enjoyment but also enhance the consumers’ intention to interact with the object. As brands are increasingly linked with hedonic consumption, therefore, the expressive aesthetic appeal should be addressed in social media advertising to develop consumer engagement with brands. Furthermore, the finding suggested that social media users respond favorably to ads that offer entertaining and trending brand relation news and brand-related offerings which foster consumers’ interaction with the brand ads. Accordingly, social media ad developers might consider developing content more amusing and funny with up-to-date brand-related offerings to create a temporary infusion of excitement that exerts a positive impact on consumer-perceived ad value as well as consumer–brand interactions.

This study has further evidenced that positively engaged customers generate positive WOM in the online social community, which increases the chances of customers’ purchase intentions and perceptions of the brand. Therefore, brands should rethink, redesign, and restrategize their social media ads in a way which are the source to engage their customers positively. The positive engagement, which leads to value co-creation, provides a distinctive edge for the brands that further develop consumer–brand relationships, driving purchase intention and positive business outcomes.

### Limitations and Agenda for Future Research

We concluded by offering an overview of key limitations that arise from this research and which offer opportunities for further investigation. First, our sample was sourced mostly from the students, resulting in potentially limited generalizability of our findings. Therefore, future research is encouraged to test and validate our model in different age groups and people from different occupations to explore how social media advertising impacts consumers’ value co-creation behavior. Second, we did not study the interactivity of- and between the consumers and the providers in terms of how they can team up in establishing value co-creation. Future research studies may wish to develop a conceptual framework highlighting the role of brands and the customers in developing potential value, value-in-exchange (value co-creation), and value-in-use (value creation) while examining the repurchase intentions and e-WOM in an online service context. Third, the value co-creation mechanism between the service firms and their customers is not fully understood and needs consideration. Future research studies should empirically examine the co-creation phenomena in terms of how the two parties accept and reject the demands of value that are claimed to be meaningful to the individual parties (brands vs. customers). Finally, this study is only confined to examining the repurchase intentions of the customers. Further researchers may wish to explore the drivers of customers’ willingness to co-create value and examine the outcomes in the form of customers’ co-creation experience in different industrial settings for instance online retailing, freemium vs. premium gaming context.

## Data Availability Statement

The raw data supporting the conclusions of this article will be made available by the authors, without undue reservation.

## Author Contributions

AH: idea generation, methodology, data analysis, and write-up. DT concept development and write-up. MM literature review. All authors contributed to the article and approved the submitted version.

## Conflict of Interest

The authors declare that the research was conducted in the absence of any commercial or financial relationships that could be construed as a potential conflict of interest.

## Publisher’s Note

All claims expressed in this article are solely those of the authors and do not necessarily represent those of their affiliated organizations, or those of the publisher, the editors and the reviewers. Any product that may be evaluated in this article, or claim that may be made by its manufacturer, is not guaranteed or endorsed by the publisher.
